# The Benefits of Licensed Midwifery and Community Birth Among BIPOC Birthing People in New Mexico

**DOI:** 10.1111/birt.12884

**Published:** 2024-12-13

**Authors:** Raquel Z. Rivera, Micaela Lara Cadena, Jess F. Gutfreund, Esperanza Dodge

**Affiliations:** ^1^ Bold Futures NM Albuquerque New Mexico USA; ^2^ Health Policy Researcher, Advocate and Birthworker New Mexico USA; ^3^ Birth Center Equity Dixon New Mexico USA; ^4^ Community Doula Albuquerque New Mexico USA

**Keywords:** community birth, direct‐entry midwives, Licensed Midwives

## Abstract

**Background:**

Black, Indigenous, and people of color (BIPOC) families comprise a disproportionately low percentage of home and freestanding birth center births in New Mexico (NM), despite NM Medicaid coverage of care by Licensed Midwives (LMs) in these settings. The purpose of this study was to examine why low income BIPOC seek out LM care, how they benefit from this model of care, and which factors facilitate and obstruct access.

**Methods:**

We conducted 7 focus groups with 55 low income BIPOC individuals who had birthed in New Mexico in the past 5 years. Participants in four of the groups intended to birth with an LM in the community setting; participants in three of the groups intended to birth in a hospital.

**Results:**

Prior negative birthing experiences at hospitals were the most‐often discussed reason for choosing LM care. The aspects of LM care most commonly described as beneficial were: (1) the high quality of one‐to‐one individualized and holistic care offered by LMs, as well as (2) the respectfulness of care received. Medicaid coverage of LM care and special payment allowances made by LMs were cited as two important facilitators of access to LM care. Barriers to care included the lack of general awareness of LM care, the persisting stigma against community birth, the small number of LMs, and payment and insurance coverage challenges.

**Conclusion:**

LM care is beneficial for many families seeking respectful and accessible care, especially in underserved areas. BIPOC birthing individuals' reflections on their experiences with LM care provide valuable information that should be considered when designing and revising perinatal care systems and policies with the intent of increasing access to high‐quality maternal and newborn care in New Mexico and, more generally, the United States.

## Graphical abstract

Black, Indigenous, and people of color (BIPOC) families comprise a disproportionately low percentage of home and freestanding birth center births in New Mexico, despite NM Medicaid coverage of care by Licensed Midwives (LMs) in these settings. The purpose of this study was to examine why low income BIPOC seek out LM care, how they benefit from this model of care, and which factors facilitate and obstruct access. This figure summarizes the factors that study participants described as the benefits of LM care, their reasons for choosing LM care, and the barriers to LM care.

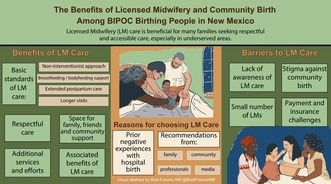

## 1 Introduction

The benefits of perinatal care by Licensed Midwives (LMs) and other direct‐entry midwives—many of whom fall under the Certified Professional Midwife (CPM) [1] credential—who practice in “community settings” (i.e., homes or freestanding birth centers) have been identified in various studies. Of particular note in previous studies are high scores on respect and autonomy indexes [2, 3], the safety of planned home births for low‐risk birthing people [4, 5, 6, 7, 8, 9], substantially lower costs [10, 11], and the potential to positively impact maternal/infant health inequities [2, 3, 12, 13, 14, 15, 16, 17].

In New Mexico, direct‐entry midwives are credentialed as LMs and have autonomous practice within broad state scope of practice guidelines. New Mexico Medicaid presently covers prenatal care, labor and delivery, and 1 year of postpartum care with LMs at home or in a birth center. (Only 6 weeks of postpartum care was covered when we conducted this research.) For comparison, across the US, only 36 states and the District of Columbia have a path to licensure for direct‐entry midwives like LMs and CPMs; while Medicaid covers births with LMs and CPMs in only 19 states [18, 19].

New Mexico's provisions regarding Medicaid coverage of LM care should presumably create an accessible landscape for low income Black, Indigenous, and people of color (BIPOC) where costs would not restrict access to midwifery care. However, BIPOC families in New Mexico comprise only 36% of home and freestanding birth center births in the state—yet represent 70% of overall births [20]. (This disproportionality is also present nationwide [21, 22, 23, 24].) The fact that these services and coverage are available does not guarantee knowledge of or access to them by most birthing people and families that could benefit from midwifery models of care.

The purpose of this study was to examine why low income BIPOC seek out LM care, how they benefit from this model of care, and which factors facilitate and obstruct access to this type of midwifery care.

## 2 Methods

Between 2016 and 2017, we conducted 7 focus groups in Española, Albuquerque, and Las Cruces, New Mexico with 55 people who had birthed in the state in the past 5 years. While the focus group participants had received perinatal care and given birth in a variety of locations (home, freestanding birth centers, hospitals), the principal intent of this research was to identify and describe the experiences of people who received care from LMs at home and in freestanding birth centers.

Four of the focus groups consisted of people who had birthed (or intended to birth) with an LM; three of the groups consisted of people who had intended to give birth in a hospital. (One of the LM focus groups inadvertently included people who birthed with CNMs. For the data analysis in the next section, we did not code this focus group.) All participants met the income threshold for the NM Women, Infants, and Children (WIC) Program (185% of the Federal Poverty Level) at the time they were pregnant; this income threshold is lower than the one for NM Pregnancy Medicaid (250% FPL). Participants identified as Hispanic or Latina (71%), American Indian or Native American (24%), African American or Black (6%), and Asian American (2%). (Percentages do not add up to 100, since multi‐racial people were counted in each of the categories they named. Multi‐racial people who identified as white were only counted in the BIPOC groups they marked.) Among participants, 19% were LGBTQ+ and 9% were single (i.e., had no partner) at the time they were pregnant. (These ethnicity/race and sexual orientation percentages do not include one of the LM focus groups, given the unfortunate loss of the de‐identified survey data).

Our team used a qualitative research design that combined grounded theory and critical inquiry. We made systematic comparisons between observations in the focus group data and categories that emerged from participants' narratives. These categories became the codes that guided the analysis and allowed the team to identify the most salient themes and ideas expressed by participants. Survey findings supplemented and contextualized the focus group data.

Low income BIPOC families were the focus of this research for several reasons: First, prior research on LMs and other midwives who practice in community birth contexts has largely focused on white, affluent populations that have had the greatest access to direct‐entry midwifery (one notable exception is the Giving Voice to Mothers [GVTM] study and the articles based on this US national survey) [2, 3]. Second, BIPOC comprise the majority of births in New Mexico. Third, BIPOC communities are disproportionately affected by health inequities [2, 3, 13, 17], which includes not having access to the kind of birth care they desire [23].

Our study focused on LMs, rather than on Certified Nurse Midwives (CNMs) or other providers who may attend community births, for two reasons: First, during the years when the team designed and conducted the research, the vast majority of community births in New Mexico were attended by LMs. CNM numbers have increased since then, whereas LM numbers have decreased. (The values were prepared by the Statistics and Epidemiology Unit of the Bureau of Vital Records and Health Statistics, NM Department of Health, on January 20, 2023.) Second, we aimed to capture experiences associated specifically with LMs given the distinctiveness of their training and model of care in the United States [1, 16], the fact that they only practice in community birth settings (i.e., they do not have access to hospital privileges in New Mexico), and due to their unique position relative to state health systems and policies which entails “specific structural barriers to their practice” [25].

A note on terminology and study demographics: While we use the acronym BIPOC in this report to foreground the severe health inequities experienced by Black and Indigenous people in the US, it is important to acknowledge that, in terms of numbers, the study's sample demographics (and, more generally, New Mexico demographics) would more accurately be described as “Latine/Hispanic, Indigenous, Black and other people of color.” Nonetheless, we intentionally use the term BIPOC here to draw attention to those made most vulnerable by systems of oppression and to advance language justice.

## 3 Results

### 3.1 How Birthing People Choose Licensed Midwives

Two main factors emerged from participants as having an impact on their decision to give birth with the care of an LM: (1) prior negative experiences of disrespect [2, 3] while birthing in a hospital context; and (2) recommendations from family, community, professionals, and media. The following quote from a participant provides an example of a negative birth experience at a hospital, which then led them to choose LM care: “During my second pregnancy, I knew I really didn't want a hospital birth. The first one was the worst experience of my life, just terrible. But I didn't know about home births. My partner and I were in a birthing class and I wondered: ‘How can I do this at home, by myself, because I do not want another hospital birth?’ It was the worst thing that ever happened to me. So, the woman who was giving the class said: ‘Here. Give this midwife a call.’” For another participant, it was her friend's positive experience with home birth, as well as a documentary and books that led her to choose LM care: “For me, it was my very good friend that I had grown up with. She had a home birth and was very much into natural birthing. I had seen her as a model and became very interested. I started doing my research, watched *The Business of Being Born*, started reading books, and then that really resonated with me and the experience that I wanted to have.”

One participant explicitly linked their decision to give birth with an LM to the longer visits typical of the LM model of care: “Just going to a doctor, they only want to talk to you for five minutes. I knew I wanted to be able to ask more questions. So, when I first met the midwife and she gave me an hour of her time, I was like, wow, this is amazing. So that's how I chose.” Longer visits were one of numerous other perceived benefits of the LM model of care that participants discussed. In the sections that follow, we describe participants' experiences of these benefits, facilitators to care with LMs, and barriers to access.

### 3.2 Benefits of Licensed Midwifery Care

Study participants discussed at length the aspects of LM care that they found most helpful and supportive, including LM's non‐interventionist approach, bodyfeeding and breastfeeding support, extended and intensive postpartum care, and longer visits both before and after birth. One participant described LMs' non‐interventionist approach this way: “For the first pregnancy, I had my baby on the Fourth of July. I've always had this feeling that maybe that's why I needed a C‐section. My doctor seemed like he was upset that he had to be there. I felt like he rushed it along; he said ‘Okay, we've got to make a decision now or your baby is potentially not going to do well.’ He never explained what was going on, or if my baby was actually breech—which she wasn't. I have seen the records and everything was fine. It seemed like it was just a ‘get this show on the road’ thing. With my home birth it was 50 hours of labor and [the midwives] were there. It was a totally different experience, mostly being able to actually labor in the tub, which was so comfortable.”

Also often discussed by participants was the respectful [2, 3] care that LMs offered. As described by participants, respect entailed support for choices, preferences and bodily autonomy. Respect was also described as contributing to feeling comfortable asking questions, declining care offered, and accepting options offered by the midwife. In the words of a participant who spoke of valuing the sense of choice she experienced: “The feeling of mutuality and not feeling like the doctor is up here and you're somewhere [down here]. And with a midwife it's more like these are your options—what do you want to do? And this is what I can help you with, and it is o.k. if you don't want to do it that way. So, it was more your plan that they're together with you on, instead of it being something you go to expect to be told what to do.”

Several additional services and efforts made by LMs that made clients feel cared for were also mentioned and deeply appreciated; including preparation of food, herbal remedies, tinctures, teas, placenta preservation, emotional support at a deep personal level, and many others. Participants also praised the inviting and inclusive space that LMs create for family, friends, and community supports. One participant described these aspects of LM care as a healing approach to birth: “[My mother] ended up being at my birth and so she got to see it all play out, and then she was like, ‘I get it.’ I thought it was a healing experience for my family and my mother. And I think that it really changed people's perceptions of possibility in my family. I think a lot of unsubstantiated fears were conquered during that time.” Overall, participants described the benefits of LM care as far beyond simply delivering a healthy baby; LM models of care, as they unfold in the community setting, nourish the entire family, help to strengthen support networks, and perhaps most importantly, offer clinical and psychosocial encounters with care providers that center healing and support—aspects of care that many who participated in this study had not experienced in other forms of health care.

### 3.3 Access to Licensed Midwives for NM Families

#### 3.3.1 Factors That Facilitate Access

Participants identified various supports for families who are seeking LM care. Many participants mentioned Medicaid coverage as key to their ability to choose home or freestanding birth center birth. One participant said: “I was part of the NAPPR program, the Native American Professional Parent Resources, and so I had a home visitor and she would come and she would help me look at options and that sort of thing. So, when I had the bad experience at the hospital, she was quick to provide other resources and I learned from her, also, that home births were covered under Medicaid even. So that's when I chose to do that.” Another participant who moved to New Mexico from another state said: “In some states [Medicaid] doesn't cover home birth. But here, coming here—I'm grateful that it covered it.”

Special payment accommodations were also described by participants as crucial to LM care access. LMs made special payment arrangements to accommodate individual family needs, such as sliding scale fees, payment plans, and prioritizing meeting in person even when the details regarding a family's insurance coverage or ability to pay were unclear; otherwise, these families would not have been able to access LM care. Participants recognized that while this is wonderful for families, it is onerous for midwives. One participant was undocumented and thus did not qualify for Medicaid. Another had lost their insurance while pregnant, and two others had insurance but would have had to pay up front. One of the participants whose insurance expected her to pay up front in order to offer reimbursement later said: “Right away I said, ‘What about payment?’ and the first thing the midwife that I chose said was, ‘Don't worry about that. Let's have our first meeting. Let's have our first appointment.’ And that really struck me, her seeing me as an individual and not just being concerned with how I was going to pay for her.”

Participants also spoke about the benefits of having access in New Mexico to both home and birth center births. While some participants preferred receiving LM care at home, others treasured the birth center as a welcome alternative to both hospital and home. The perceived extra responsibilities of birthing at home (e.g., preparing the space and having to clean up afterward) and the sense of community at a birth center are two reasons why birthing people might prefer to receive LM services at a freestanding birth center rather than at home. In the words of one participant: “For me, at the birthing center I chose, it felt very community‐like. So, thinking back to my first pregnancy, I walked into the office of the OB/GYN, and I sat for like an hour or more before I got called back with all of these other pregnant ladies that I felt a connection with but there was no permission to say, ‘Hey, give me a call,’ or whatever. But at the birthing center, even seeing some of these friendly faces in the room, it was just like, ‘Hey.’ It was a community kind of area. So that's, for me, what was positive about using a midwife through my pregnancy—that community and being with other moms.”

#### 3.3.2 Factors That Obstruct Access

In New Mexico, many families do not know that LMs are licensed by the state and covered by Medicaid. Participants who received LM care expressed frustration that most families are still unaware that this option exists. Out of 23 study participants interviewed who had not received LM care, only two knew of its existence and that it was covered by Medicaid. Some participants described wishing they had known earlier that LM care was a quality option covered by Medicaid.

Stigma against home birth is another barrier to LM care in New Mexico. Focus group participants who received LM care repeatedly expressed that stigma against home birth made it harder to choose and harder to remain confident in that choice. Giving birth outside of the hospital was commonly questioned by family and friends, as well as professionals or acquaintances, who argued against the wisdom of participants' pregnancy and birth care choices. In the words of one participant: “Our family, just, everybody thinking the same thing. ‘What if something happens? What are you going to do? Can't you just have it at the hospital?’ And then our coworkers, because I do know a lot of nurses, and so it was like me telling them that I was going to Mars or something, every time I told them that I was going to have a home birth. And even now, whenever birth comes up and I mention that I had a home birth, everybody's jaw just drops and they look at me like I'm crazy or something.” Another participant experienced having providers try to dissuade her from having a community birth: “There was one time when I was trying to contact or find a midwife. The actual clinics, they were against it, and they were trying to convince me not to do it or to speak about it.”

Another challenge to LM care access is the small number of LMs throughout the state; LMs are especially scarce in certain rural regions [25, 26], and BIPOC LMs are even more so. Some participants expressed a strong preference for LMs who shared their racial, ethnic, or cultural identity, yet the small number of BIPOC LMs made this preference for cultural congruence difficult or unviable. As expressed by a Black participant who spoke of having experienced blatant racism at a local hospital: “It was really important for me to find a midwife of color after those experiences I had. And then, calling around, even the birthing center, I think they had one person of color, and then I couldn't meet their other expectations either. So, I got connected to [a midwife of color] from someone else who had used her. That was, I think, a little bit of a lucky shot.”

There are also various challenges to accessing LM care related to payment and insurance coverage issues. A major barrier is the inaccessibility of Medicaid coverage for undocumented pregnant people. Meanwhile, for those with private insurance coverage, a slow insurance approval process and/or out‐of‐pocket payments present major challenges to clients hoping to utilize LM care as many pregnant people cannot afford to pay up front or engage in a payment plan. The unexpected withdrawal of insurance coverage is also a barrier, negatively impacting birthing individuals, and sometimes having serious consequences for pregnancy outcomes. One participant who lost their insurance during their pregnancy described that loss as highly stressful and as contributing to a stress‐related health condition that threatened their ability to give birth with an LM in the community setting. The out‐of‐pocket birth center facility fee not covered by Medicaid was another payment‐related challenge described by participants. At the time data were collected for this study, some freestanding birth centers charged a self‐pay facility fee because Medicaid did not cover facility‐related costs for birth centers.

## 4 Discussion

Birthing people's perceptions, desires, experiences, and satisfaction regarding birthing and perinatal care are integral, not peripheral, to the provision of quality, person‐centered care [27] and are critical for improving both short‐ and long‐term health outcomes [28, 29]. Thus, the insights that study participants shared regarding the benefits of LM care highlight the value and importance of LMs for many in our communities, and also illustrate how hospitals could benefit from creatively assessing ways to integrate ideas from LM care and even community‐based LM care into their health systems [9, 25, 30].

Medicaid coverage of LM care must be as consistent, dependable, and expedient as possible for both LMs and people with Medicaid eligibility. New Mexico Medicaid and Medicaid managed care organizations (MCOs) must have clear lines of communication with all entities, members and stakeholders in order to facilitate the processes needed to access community‐based care with a licensed midwife. Only approximately half of LMs participate in the NM Medicaid Birthing Options Program [26] which includes home and freestanding birth center birth under the care of LMs; this limited engagement of LMs with the program severely restricts widespread access to community midwives and is rooted in the difficulties experienced by LMs engaging with the program [25, 31]. Furthermore, whenever new laws or rules pertaining to LM services are proposed, careful consideration must be given to ensure access to both home and freestanding birth center births. It is also important that New Mexicans be truly informed about all their care options under the NM Medicaid's Birthing Options Program. Information sheets could be sent out to each pregnant Medicaid client, detailing all the care options covered, and private insurance clients should be offered the equivalent. Such an approach will not only help address low awareness about LM care, but also persisting stigma and misinformation.

New Mexico has an opportunity to improve access to quality care for families, and to make the midwife profession more sustainable for those who practice it. The state must prioritize policy pursuits that represent the best interests of BIPOC communities who are impacted by under‐resourced healthcare landscapes and perinatal health inequities. A crucial part of those pursuits is the development of a diverse workforce that can provide the culturally concordant care necessary for improving birth outcomes [9, 14, 32, 33, 34, 35].

Future comparative research is needed to better understand how to remove barriers to community‐based licensed midwifery care for families that desire it, not only in New Mexico but also in other states. Since some of these barriers are directly related to provider shortage, limited engagement of LMs with Medicaid and workforce diversity issues [25, 31], priority must be given to research that investigates the experience of LMs and other credentialed community‐based providers (i.e., Certified Nurse Midwives and Certified Professional Midwives) and explores the difficulties related to entering and sustaining the practice—particularly for BIPOC midwives.

## 5 Conclusion

Low income BIPOC birthing individuals who have received LM care in New Mexico seek out and value this care for many reasons, including: (1) the high quality of one‐to‐one individualized and holistic care offered by LMs, as well as (2) the respectfulness of care received compared to the care offered in hospital settings. The opportunity for birthing individuals to receive intensive support from family and friends while receiving LM care and the additional services and support offered by LMs that are not offered in hospitals (such as herbal remedies, placenta preservation, longer visits, and in‐depth emotional support) are also among the other perceived benefits of receiving LM care.

People who have received LM care in New Mexico recognize and appreciate the increased access to LM care possible in the state, given that LM licensure exists and Medicaid coverage is available. However, participants were also concerned over the many barriers that still exist to accessing LM care, including a lack of awareness by many individuals and families regarding LM care, the stigma still surrounding home and freestanding birth center birth, the small number of LMs throughout the state (particularly in many rural areas), the even smaller number of BIPOC LMs and various payment and coverage challenges, both for Medicaid and private insurance clients.

The experiences of New Mexico families and birthing people detailed in our study deserve careful consideration when designing systems and policies related to perinatal care, as well as when reviewing and revising these systems and policies.

## Ethics Statement

The project received approval from the University of New Mexico Main Campus IRB.

## Conflicts of Interest

The authors declare no conflicts of interest.

## Data Availability

Given the relatively small numbers of people receiving Licensed Midwifery care as well as the small numbers of Licensed Midwives providing care in New Mexico, we will not be sharing data to prevent potential breaches of confidentiality.
